# Research progress on the oligosaccharide components and pharmacological activities of *Morinda officinalis*

**DOI:** 10.3389/fchem.2026.1813259

**Published:** 2026-03-31

**Authors:** Yan Lei, Zhaohan Wang, Yuxian Dan, Yongxian Xie, Yongheng Wang, Xinluan Wang, Li Zhang

**Affiliations:** 1 College of Traditional Chinese Materia Medica, Shenyang Pharmaceutical University, Shenyang, China; 2 Institute of Traditional Chinese Medicine and Natural Products, College of Pharmacy, State Key Laboratory of Bioactive Molecules and Druggability Assessment, International Cooperative Laboratory of Traditional Chinese Medicine Modernization and Innovative Drug Development of Chinese Ministry of Education of China, Guangdong Province Key Laboratory of Pharmacodynamic Constituents of TCM and New Drugs Research, Jinan University, Guangzhou, China; 3 Shenzhen Institutes of Advanced Technology, Chinese Academy of Sciences (CAS), Shenzhen, China; 4 The Second Affiliated Hospital of Guangzhou University of Chinese Medicine, (Guangdong Provincial Hospital of Chinese Medicine), Guangzhou, China

**Keywords:** Morinda officinalis, oligosaccharides, separation, pharmacological activity, research progress

## Abstract

Morinda officinalis, a traditional Chinese medicine, is an authentic medicinal herb from Deqing, Guangdong, and is also one of China’s famous “Four Great Southern Medicinals”. It is widely used as a dietary supplement in the southern regions and is known for its effects on kidney yang, as well as strengthening bones and muscles. The main active components of *Morinda officinalis* include sugars, anthraquinones, and iridoids. Among these, sugars-including monosaccharides, polysaccharides, and oligosaccharides-are considered the primary bioactive constituents. In recent years, pharmacological studies have indicated that the oligosaccharide components of *Morinda officinalis* possess significant pharmacological activities, such as antidepressant, anti-Alzheimer’s, and anti-osteoporosis effects, highlighting their potential for further development and utilization. Therefore, this paper reviews the separation processes and pharmacological activities of oligosaccharide components in *Morinda officinalis*, and summarizes the research and application hotspots of *Morinda officinalis* oligosaccharides, aiming to provide a theoretical basis for further research.

## 1 Introduction

*Morinda officinalis* How is the dried root of *Morinda officinalis* which belongs to Rubiaceae, has a slight aroma and a sweet, slightly astringent taste. In 1958, after investigation and identification by Professor Kuan-zhao Hou, the medicinal plant of *Morinda officinalis* was named *Morinda officinalis* How. It has been included in the *Pharmacopoeia of the People’s Republic of China* (hereinafter referred to as the *Chinese Pharmacopoeia*). The 2000 edition of the *Chinese Pharmacopoeia* describes *Morinda officinalis* as the dried root of *Morinda officinalis* How (family Rubiaceae, genus Morinda), and it has been officially recognized since then. It is artificially cultivated for medicinal use in the Chinese provinces of Fujian, Guangdong, Guangxi, and Hainan (Dai et al., 2021; Lin et al., 2010).

*Morinda officinalis* was first recorded in the classic text *Shen Nong Ben Cao Jing* and was classified as a top-grade herb. According to the *Chinese Pharmacopoeia*, it is known for its effects on invigorating kidney yang, strengthening bones and muscles, and dispelling dampness and wind. It is used to treat conditions such as impotence, nocturnal emission, cold uterus, infertility, and irregular menstruation. Traditional Chinese Medicine (TCM) theory holds that the kidney is the foundation of innate constitution. The kidney stores essence, governs bone development, and produces marrow, which connects to the brain. Human vision, hearing, smell, sensation, as well as thinking and memory, all originate from the brain. Therefore, TCM adopts the principle of “treating based on kidney theory, tonifying the kidney and replenishing essence, supplemented by promoting blood circulation to remove stasis, and removing turbidity and resolving phlegm” to treat dementia. Thus, in addition to the above traditional effects, *Morinda officinalis* also exhibits pharmacological effects such as antidepressant, anti-Alzheimer’s, anti-osteoporosis, antioxidant, and immune-enhancing properties. In March 2002, the National Health Commission of China issued a notice on further regulating the management of health food raw materials, listing *Morinda officinalis* as a Chinese medicinal material suitable for use in health foods. At present, based on the traditional efficacy and pharmacological effects of *Morinda officinalis*, a series of *Morinda officinalis*-based drugs have been developed, such as *Morinda officinalis* oligosaccharides capsules, *Morinda officinalis* kidney-tonifying pills, and nourishing ointments. Utilizing its ingredients, which are rich in vitamin C, sugars, and essential amino acids, it has been used in healthcare products and dietary supplements, such as *Morinda officinalis*-steamed chicken and *Morinda officinalis* beverages (Song et al., 2018).

**FIGURE 1:**
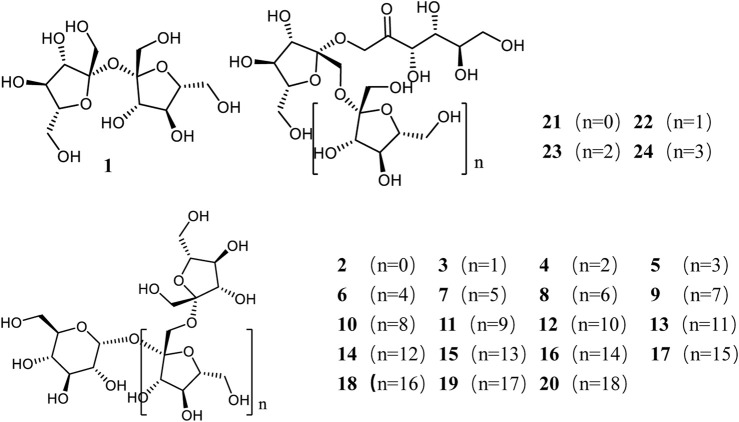
Structural formula of oligosaccharide components in *Morinda officinalis.*

The main chemical constituents of *Morinda officinalis* include sugars, anthraquinones, iridoids, and organic acids. Among them, sugars are the major constituents. The 2025 edition of the *Chinese Pharmacopoeia* records that the oligosaccharide-nystose is the quality control component of *Morinda officinalis* and stipulates that its content shall not be less than 2.0%. At present, 24 kinds of oligosaccharides have been extracted and isolated from *Morinda officinalis*, including Bajijiasu, sucrose, kestose, nystose, 1F-fructofuranosyl nystose, and inulobiose to inulin eicosasaccharide (Table 1). These can be classified into two major categories: one composed of glucose and fructose (inulin-type oligosaccharides, with the general formula α-D-glucopyranose-[(2→1)-β-D-fructofuranosyl-(2→1)]_n_-β-D-fructofuranose), and the other composed of fructose (inulo-oligosaccharides, with the general formula β-D-fructopyranose-[(2→1)-β-D-fructofuranosyl-(2→1)]_n_-β-D-fructofuranose) (Figure 1) (Hao et al., 2018).

**TABLE 1:** *Morinda officinalis* oligosaccharides.

No.	CAS Name/English name	Chemical formula	CAS number
1	Bajijiasu	C_12_H_22_O_11_	847661-51-2
2	Sucrose	C_12_H_22_O_11_	57-50-1
3	Kestose	C_18_H_32_O_16_	562-68-5
4	Nystose	C_24_H_42_O_21_	13133-07-8
5	1F-fructofuranosyl nystose	C_30_H_52_O_26_	59432-60-9
6	Inulin hexasaccharide	C_36_H_62_O_31_	—
7	Inuloheptaose	C_42_H_72_O_36_	612838-82-1
8	Inuloctaose	C_48_H_82_O_41_	—
9	Inulononaose	C_54_H_92_O_46_	697278-39-0
10	Inulodecaose	C_60_H_102_O_51_	697278-40-3
11	Inulin undecaose	C_66_H_112_O_56_	—
12	Inulin dodecaose	C_72_H_122_O_61_	—
13	Inulin tridecaose	C_78_H_132_O_66_	—
14	Inulin tetradecaose	C_84_H_142_O_71_	—
15	Inulin pentadecaose	C_90_H_152_O_76_	—
16	Inulin hexadecaose	C_96_H_162_O_81_	—
17	Inulin heptadecaose	C_102_H_172_O_86_	—
18	Inulin octadecaose	C_108_H_182_O_91_	—
19	Inulin enneacontaose	C_114_H_192_O_96_	—
20	Inulin eicosasaccharide	C_120_H_202_O_101_	—
21	Inulobiose	C_12_H_22_O_11_	470-58-6
22	Inulotriose	C_18_H_32_O_16_	58208-59-6
23	Inulotetraose	C_24_H_42_O_21_	58208-60-9
24	Inulopentaose	C_30_H_52_O_26_	74903-83-6

The nystose content of *Morinda officinalis* oligosaccharides determines the quality control standards for *Morinda officinalis*. Nystose is also the main medicinally active component of *Morinda officinalis*, exhibiting strong pharmacological activities, including antidepressant, anti-Alzheimer’s, anti-osteoporosis, antioxidant, and angiogenesis-promoting effects. These properties contribute to the high development potential of products, such as *Morinda officinalis* oligosaccharide capsules, which are widely used in clinical applications (Lu et al., 2018). The content and type of *Morinda officinalis* oligosaccharides directly affect their effectiveness and safety in clinical applications. However, there is currently a lack of systematic reviews on the separation and pharmacological efficacy of *Morinda officinalis*. Therefore, this paper focuses on *Morinda officinalis* oligosaccharides, providing a comprehensive review of their separation, purification, and pharmacological activities from 1995 to 2024, aiming to offer references for the development and clinical applications of *Morinda officinalis* oligosaccharides.

## 2 Separation and purification

Common separation methods for oligosaccharides include column chromatography and preparative chromatography. Among these methods, macroporous resin column chromatography is widely used for the separation and purification of *Morinda officinalis* oligosaccharides, whereas preparative chromatography is typically used to isolate individual oligosaccharide components.

Cheng-bin Cui et al. (1995) first isolated and identified four water-soluble oligosaccharide monomers from *Morinda officinalis*, marking the first time these compounds were obtained from plants of the genus Morinda. These include nystose, 1F-fructofuranosyl nystose, inulin hexasaccharide and inuloheptaose (Yang et al., 2008).

Jian-hua Zhang et al. (2018) reported that multiple oligosaccharides were isolated from the roots of *Morinda officinalis*, including Bajijiasu, nystose, 1F-fructofuranosyl nystose, inulotriose, inulotetraose, inulopentaose. Among them, sucrose, kestose, nystose, and 1F-fructofuranosyl nystose are often used as the basis for quality control of *Morinda officinalis* medicinal materials (Chen, 2022).

Zhen-zhen Huang (2013) first attempted to use acetylation-deacetylation chromatography to separate oligosaccharide from *Morinda officinalis* and used ESI-MS, ^1^H-NMR, ^13^C-NMR, and other spectroscopic methods to identify their structures, isolating three oligosaccharides: kestose, nystose, and 1F-fructofuranosyl nystose.

Qing-xiu Hao et al. (2018) investigated the rapid identification of oligosaccharides in *Morinda officinalis* using UPLC-Q-TOF-MS^E^ technology. The authors identified 19 oligosaccharide components from *Morinda officinalis*, mainly including fifteen newly reported oligosaccharides ranging from inulin hexasaccharide to inulin eicosasaccharide.

## 3 Pharmacological activity

### 3.1 Antidepressant activity

Depression is a common mental illness, characterized by low mood, slowed thinking, and psychomotor retardation as typical symptoms. Moreover, studies have shown that depression has one of the highest incidence and disability rates worldwide. Animal studies, cellular experiments, and human clinical trials have demonstrated that the oligosaccharides of *Morinda officinalis*, rather than its polysaccharides, are the principal active components.

Cheng-bin Cui et al. (1995) first discovered the antidepressant activity of the traditional Chinese medicine *Morinda officinalis* and its monomers. This discovery also provided the first examples of oligosaccharides with antidepressant activity. Ling-zhi Xu et al. (2017) tested the antidepressant effects of *Morinda officinalis* oligosaccharides using two depression models: chronic unpredictable stress and forced swim test. They found that *Morinda officinalis* oligosaccharides exerted antidepressant effects during treatment. Moreover, the results indicated that the brain-derived neurotrophic factor (BDNF)-glycogen synthase kinase-3β (GSK-3β)-β-catenin pathway in the medial prefrontal cortex might mediate the antidepressant effects and stress recovery capacity of *Morinda officinalis* oligosaccharides.

*Morinda officinalis* oligosaccharide capsules (MOO capsules) mainly contain inulin-type oligosaccharides extracted from the roots of *Morinda officinalis*. Pharmacological studies have shown that MOO capsules can improve the symptoms of depression and dysphoria by increasing 5-hydroxytryptamine (5-HT) levels in the brains of mice and enhancing the expression of brain-derived neurotrophic factors in young rats. Zheng-wei Zhang et al. (2022) revealed for the first time that MOO could alleviate depression by increasing 5-hydroxytryptophan (5-HTP) levels in the gut microbiota. This finding demonstrated that MOO exerted antidepressant effects by modulating the tryptophan/5-hydroxytryptophan (5-HTP) metabolic pathway in the gut microbiota.

Li-xuan Yang et al. (2023) demonstrated that *Morinda officinalis* oligosaccharides exert both antihypertensive and antidepressant effects. They found that these oligosaccharides could inhibit inflammation and astrocytes damage in hypertension combined with depression, by inducing mitophagy in mitochondrially damaged inflammatory astrocytes and up-regulating the expression of Mfn2 in astrocytes.

Recently, numerous researchers have investigated the antidepressant molecular mechanism of *Morinda officinalis* using network pharmacology and the Traditional Chinese Medicine Integrated Pharmacology Computing Platform (TCMIP), leading to the construction of a comprehensive visual network of TCM-component-target-pathway interactions. Research findings indicate that the primary pharmacological targets are 5-HT receptors 2A, 2B, and 2C. The mechanism may involve reducing neuronal damage in the hippocampus and oxidative damage in brain tissue, directly enhancing the expression of 5-HT neurotransmitters, increasing the expression of brain-derived neurotrophic factor (BDNF), and regulating hippocampal neuroplasticity. Collectively, *Morinda officinalis* demonstrates multifaceted antidepressant activity through multiple components, targets, and pathways, primarily modulating the complex interactions between neural, immune, and endocrine systems (Deng et al., 2021; Lang et al., 2019; Wei and Yue, 2017).

### 3.2 Anti-Alzheimer’s activity

Alzheimer’s disease (AD) is a neurodegenerative disease, mainly manifested by progressive memory loss, cognitive dysfunction, and personality changes. The global population aging trend is projected to triple the number of AD patients. Studies have shown that *Morinda officinalis* oligosaccharides are prebiotics that improve memory in animal models of AD (Yang et al., 2018; Chen, 2012).

Di-ling Chen et al. (2013a) demonstrated that *Morinda officinalis* oligosaccharides significantly improved the learning and memory ability of Aβ_25-35_ (β-amyloid peptide) dementia-mimicking model rats. Their mechanism of action may involve enhancing monoamine neurotransmitter levels, inhibiting neuronal apoptosis, increasing antioxidant capacity, activating cerebral energy metabolism, and ameliorating cholinergic system damage, thereby alleviating the effects of Alzheimer’s disease.

Yang (2016) investigated the protective mechanisms in AD models, demonstrating significant neuroprotective effects against Aβ_25-35_-induced injury in primary cultured rat astrocytes. The protective mechanism was primarily mediated through activation of the Nrf2/ARE (Nuclear factor erythroid 2-related factor 2/Antioxidant Response Element) signaling pathway, as evidenced by the upregulation of Nrf2, HO-1 (Heme Oxygenase-1), NQO1 (NAD (P) H: Quinine Oxidoreductase 1), and *p38* mRNA, as well as downregulation of GSK-3β (Glycogen Synthase Kinase-3β), and Caspase-3. These molecular changes directly or indirectly inhibited cellular injury, thereby exerting a protective effect on AD cell injury models.

Di-ling Chen (2012) conducted a study on the preparation process and qualitative and quantitative identification of bajijiasu. Based on this, a combined *in vitro* and *in vivo* approach using cellular and animal models was used. At the cellular and molecular levels, it was revealed that bajijiasu improves the antioxidant capacity by lowering intracellular calcium ions and inhibiting the activation of pro-modulatory factors such as NF-kB (Nuclear Factor kappa-light-chain-enhancer of activated B cells) and JAK2/STAT5 (Janus Kinase 2/Signal Transducer and Activator of Transcription 5). Meanwhile, it activates P21 and inhibits the expression of cyclic regulatory proteins like CDK4 (Cyclin-Dependent Kinase 4) and E2F1, thereby suppressing neuronal cell death. Animal experiments further demonstrated that *Morinda officinalis* can ameliorate cholinergic system impairment by activating cerebral energy metabolism, thus alleviating the symptoms of Alzheimer’s disease.

Related studies have shown that kestose, a component in MOO, has memory-improving effects in AD model animals and is considered potential prebiotic. Di-ling Chen et al. (2017) explored the prebiotic effects of kestose in MOO on Alzheimer’s disease by targeting the microbial-gut-brain axis in rodent models. They suggested that the microbial-brain-gut axis could be used as a potential new therapeutic target, providing new research directions for the treatment of various neurological diseases such as AD.

### 3.3 Anti-osteoporotic activity

Osteoporosis (OP) is a systemic, metabolic bone disease. Clinically, traditional Chinese medicine offers unique advantages in OP treatment (Yang et al., 2018). Although polysaccharides and monotropein have been widely studied as active components of *Morinda officinalis*, research focusing on the relationship between its oligosaccharide components and OP remains limited (Yang et al., 2024).

Su-yu Zheng (2013) and Peng Zhang (2014) demonstrated that *Morinda officinalis* oligosaccharides significantly improve postmenopausal osteoporosis by increasing bone mineral density and serum OPG levels while inhibiting RANKL expression, thereby effectively preventing and treating postmenopausal osteoporosis (PMOP). Subsequent study by Hao-yan Chen (2021) investigated the therapeutic effects of *Morinda officinalis* on PMOP and found that it exerts anti-osteoporotic effects by regulating the OPG/RANKL signaling axis, leading to increased bone mass, improved trabecular morphology and bone microarchitecture, and enhanced bone metabolic balance.

Wen-de Zhuang et al. (2022) investigated the effects of different processing methods of *Morinda officinalis* oligosaccharides on oxidative stress and femoral tissue morphology in a mouse model of cyclophosphamide-induced osteoporosis. They focused on the following oligosaccharide components: D-fructose, sucrose, 1-kestose, nystose, and 1F-fructofuranosyl nystose and found that these oligosaccharides can improve oxidative stress indicators, and low-dose *Morinda officinalis* oligosaccharides exert a more significant ameliorative effect on cyclophosphamide-induced osteoporosis.

### 3.4 Other pharmacological activities

Jun Zhao et al. (2018) investigated the mechanism by which MOO enhances fertility in infertile mice. They proposed that MOO might act through regulating the function of the pituitary-hypothalamus-gonadal axis to control the process. Meanwhile, it enhances the activity of antioxidant enzymes *in vivo*, playing an antioxidant role to protect testicular spermatozoa from oxidative damage. Jing-ke Yang (2007) studied the role of *Morinda officinalis* oligosaccharides in promoting therapeutic angiogenesis, and reported that they significantly promote angiogenesis in the chick embryo chorioallantoic membrane and in the ischemic myocardium of rats with acute myocardial infarction (AMI). Furthermore, they increase the expression of VEGF and bFGF proteins in ischemic myocardium, suggesting a possible mechanism for their pro-angiogenic effects. Chao-dou Xu et al. (2003) investigated the pro-immunoactivity effects of *Morinda officinalis* oligosaccharides. However, their mechanism of action remains to be further studied. Kui-peng Wang (2007) reported that *Morinda officinalis* oligosaccharides have a significant promoting effect on the proliferation and differentiation of rat myoblasts, and can increase the expression of TGF-β1 (transforming growth factor-β1) in these cells within a certain concentration range. Studies by Bao-jun Wang et al. (2010) have shown that *Morinda officinalis* oligosaccharides ameliorate myocardial ischemia-reperfusion injury in rats. Their mechanism of action may involve increasing the activity of Na^+^-K^+^-ATPase, Ca^2+^-ATPase, and Mg^2+^-ATPase, thereby exerting myocardial protective and antioxidant effects.

The research on the pharmacological activities of *Morinda officinalis* mentioned above is summarized in Table 2.

**TABLE 2:** The biological activities of *Morinda officinalis*.

Pharmacological activity	Bioactive components of MO	References
Antidepressant	MOs	Xu et al., (2017); Zhang et al., (2022); Yang et al., (2023)
	Nystose, 1F-fructofuranosyl nystose, inulin hexasaccharide, inuloheptaose	Cui et al., (1995)
Anti-Alzheimer	MOs	Chen, (2012) Chen et al. (2013b) Yang et al. (2018)
	Bajijiasu	Chen, (2012)
Anti-osteoporosis	MOs	Zheng, (2013) Zhang, (2014) Chen, (2021) Zhuang et al. (2022)
Antioxidant	MOs	Zhao and Yang (2018)
Promote angiogenesis	MOs	Yang, (2007)
Pro-immunoactivity effects	MOs	Xu et al., (2003)
Antifatigue and neuroprotection	IOMO	Zhang, (2023)

MO, *morinda officinalis*; MOs, *Morinda officinalis* oligosaccharides; IOMO, Inulin-type oligosaccharides of *morinda officinalis*.

## 4 Conclusion

*Morinda officinalis*, recognized as one of China’s “Four Great Southern Medicinal Herbs” is extensively utilized in both clinical therapeutics and functional food industries. Its main chemical components include sugars, anthraquinones, iridoid compounds, and organic acids. Among them, the oligosaccharide components exhibit strong pharmacological activities, including antidepressant, anti-Alzheimer’s, anti-osteoporosis, proangiogenesis and reproduction-enhancing effects. At present, due to their antidepressant pharmacological activities, *Morinda officinalis* oligosaccharide capsules not only have a significant antidepressant effect when used alone but also offer greater advantages when used in combination with other drugs. They can reduce adverse drug reactions, act as a synergistic agent, and effectively improve the patients’ quality of life (Pan et al., 2024; Wang et al., 2017; Ma et al., 2022; Jing et al., 2014; Cao et al., 2020).

*Morinda officinalis* contains abundant oligosaccharide components with strong pharmacological effects. The current quality standard uses only nystose as an indicator, making it difficult to accurately evaluate the intrinsic quality of the medicinal material. Additional evaluation indicators should be considered to assess the quality of the medicinal material more comprehensively. Most literature uses high-performance liquid chromatography (HPLC) for content determination. Considering the high-water solubility of oligosaccharides and their longer retention times on sugar and amino columns, using HPLC with sugar columns or amino columns for content determination is a feasible approach (Duan et al., 2024).

Current research on *Morinda officinalis* mainly focuses on pharmacological effects and chemical component analysis, especially its potential antidepressive, anti-osteoporotic, and anti-inflammatory properties. These aspects may become hot topics in future *Morinda officinalis* research. Some pharmacological studies are still in the experimental stage, and further clinical research is required (Liu et al., 2024).

The pharmacological effects of traditional Chinese medicine are characterized by a “multi-component, multi-target, and multi-pathway” mode of action. Therefore, when studying the oligosaccharide components of *Morinda officinalis*, the synergistic effects of multiple components and pathways should be considered, rather than focusing solely on individual monomers. Both oligosaccharides and polysaccharides from *Morinda officinalis* exhibit strong pharmacological activities. Oligosaccharides demonstrate prominent effects in neuroprotection and antidepressant activities, while polysaccharides are more extensively studied for their antioxidant, anti-fatigue, and immunomodulatory effects. Therefore, the research and application prospects for the chemical components of *Morinda officinalis* are broad.

Although numerous studies on *Morinda officinalis* have been conducted to date, there are still many challenges such as insufficient exploration of its chemical components, imperfect quality control standards, and a lack of in-depth research on its pharmacological mechanisms, necessitating further investigation. Therefore, this article systematically reviews the separation, purification, and pharmacological activities of *Morinda officinalis*, providing a reference for further research on this Chinese medicinal herb.
